# Mini Review: The Forensic Value of Heat Shock Proteins

**DOI:** 10.3389/fmed.2021.800100

**Published:** 2022-01-10

**Authors:** Julian Prangenberg, Elke Doberentz, Anthea Mawick, Burkhard Madea

**Affiliations:** Institute of Legal Medicine, University Hospital Bonn, Bonn, Germany

**Keywords:** heat shock proteins, immunohistochemistry, gene expression, forensics, legal medicine

## Abstract

Forensic pathologists are routinely confronted with unclear causes of death or related findings. In some instances, difficulties arise in relation to questions posed by criminal investigators or prosecutors. Such scenarios may include questions about wound vitality or cause of death where typical or landmark findings are difficult to ascertain. In addition to the usual examinations required to clarify unclear causes of death or address specific questions, immunohistochemistry and genetic analyses have become increasingly important techniques in this area since their establishment last century. Since then, many studies have determined the usefulness and significance of immunohistochemical and genetic investigations on cellular structures and proteins. For example, these proteins include heat shock proteins (Hsp), which were first described in 1962 and are so called based on their molecular weight. They predominantly act as molecular chaperones with cytoprotective functions that support cell survival under (sub) lethal conditions. They are expressed in specific cellular compartments and have many divergent functions. Central family members include, Hsp 27, 60, and 70. This mini review investigates recent research on the Hsp family, their application range, respective forensic importance, and current limitations and provides an outlook on possible applications within forensic science.

## Introduction

Molecular chaperones are present in all cells and compartments of the body and contribute to protein biosynthesis (1–3), newly synthesized protein folding, and transport to sites of action (2, 4). Heat shock proteins (Hsps) are one such family of chaperones; they are highly conserved, and their expression is increased after heat exposure (5). Increased protein expression is also linked to responses to various stimuli, including hypo- or hyperthermal conditions, oxidative stress, energy deficiency, or ischemia (6–9). Hsp nomenclature refers to the respective molecular weight; therefore, Hsp70 weighs 70 kDa. Well-known family members include, Hsp27, Hsp60, and Hsp70, which are expressed in different cellular compartments with different functions. In forensic science, Hsp70 has been used as a marker of cellular stress upon heat exposure of burn victims and in tissues of the upper respiratory tract, lungs, and kidneys (10, 11). However, detailed studies on Hsp expression in other forensically relevant areas are scarce. Therefore, this mini review addresses current immunohistochemical research on Hsps, their scope, respective forensic relevance, and current limitations. Finally, the review provides an outlook on potential future applications.

## Materials and Methods

We reviewed Medline (https://pubmed.ncbi.nlm.nih.gov) for studies published between January 1, 2000 and the September 30, 2021, for Hsp research in a forensic context. For methodology and reporting, we used the updated 2020 Preferred Reporting Items for Systematic Reviews and Meta-Analysis guidelines (12). The words “Heat Shock Proteins” and “Forensic” were used to identify studies examining Hsp research in a forensic context. The following Medical Subject Heading combination terms and Boolean operators were applied during our search: “Heat Shock Proteins” AND “Forensic.” Bibliographies of selected articles were manually reviewed for further studies. Two authors (J.P. and A.M.) independently conducted eligibility assessments and managed data extraction. Only original research articles on human specimens published in English or German were considered for review. Article eligibility was determined based on the screening of titles and abstracts.

## Results

The initial search identified 126 studies. After screening titles, 44 studies remained for further review. After reviewing abstracts and checking for availability in English or German, 27 studies were further excluded. After manually searching bibliographies, three additional articles that matched the criteria were captured. The search eventually identified 20 eligible studies conducted between 2000 and 2021. Thus, over this period, an average of 1.11 Hsp forensic studies were published per year. Most studies were published in the years, 2006, 2012–2014, and 2017 (10%, *n* = 2 each). A small majority of studies addressed fire, hypothermia, and sudden infant death syndrome (SIDS)/peripartum deaths (15%, *n* = 3 each), followed by cardiac deaths, traumatic injuries, excited delirium (ED), and drowning (10%, *n* = 2 each). Three other studies examined acute lung injury, Hsp detection in formalin-fixed human brain tissue, and methamphetamine intoxication. One study addressed traumatic injury, asphyxia, and sudden cardiac death.

Hsp70 was the most frequent study subject (95%, *n* = 19), followed by Hsp27 (25%, *n* = 5). Hsp60 and Hsp90 were investigated in two studies each. In addition, single studies dealt with Hsp72 and Hsp110. Approximately one third (30%, *n* = 6) of the selected studies were published in the International Journal of Legal Medicine, and one quarter (25%, *n* = 5) were published in Forensic Science International. Fourteen studies used a two-group design, five used multiple-group designs, and one used a one-group design. All studies had a combined total of 1,223 study specimens and 1,178 control specimens. An overview of the identified heat shock proteins, their coding genes and their respective potential applications is shown in Table 1.

**Table 1:** Overview of mentioned heat shock proteins and their respective potential applications.

**Heat shock protein**	**Coding gene**	**Potential field of application**
27	HSPB	Perinatal hypoxia/ischemia, fire deaths, hypothermia, SIDS
60	HSPD1	Traumatic injury, mechanical asphyxiation, sudden cardiac death
70	HSPA1A, A1B, A2, A7, A8, A12B and A13 (Hsp70 member 1A, 1B, 2, 7, 12B, and 13, respectively)	Drowning, drug abuse, myocardial ischemia and sudden cardiac death, fire deaths, hypothermia, excited delirium, traumatic injury, acute lung injury, mechanical asphyxiation, SIDS, formalin fixation
90	HSP90A and HSP90B	Perinatal hypoxia/ischemia, traumatic injury, asphyxiation, sudden cardiac death
110	HSPH1	Traumatic injury, asphyxiation, sudden cardiac death

## Discussion

### Cardiac Death

A polymorphic study on the HSPA1B gene (rs3036297), encoding Hsp70 (member 1B), in the Chinese population reported that individuals with an insertion allele had a comparatively lower risk of sudden cardiac death compared with individuals with a deletion allele. Thus, it was hypothesized that the rs3036297 variant regulated HSPA1B expression *via* microRNA binding and HLA-DRB5 expression *via* long-range promoter interactions, thereby contributing to a susceptibility to sudden cardiac death. Therefore, rs3036297 is a potential marker for the molecular diagnostics and genetic counseling of sudden cardiac death (13).

In blood samples collected up to the first day after an acute myocardial infarction and analyzed by enzyme-linked immunosorbent assay, Hsp70 levels were reportedly twice as high as in control patients with angina. Moreover, peak Hsp70 levels 6 h after infarction correlated significantly with creatine kinase and cardiac troponin T levels, as well as with interleukin-6 (IL-6) and IL-8. Thus, circulating Hsp70 could be a suitable marker of myocardial damage and may play a role in inflammatory responses after acute myocardial infarction (14).

### Fire

Hsp70 was identified as a reliable marker for the antemortem impact of fire or hot vapor inhalation. Protein expression was significantly increased in the epiglottis, trachea, main bronchi, and peripheral bronchi of burn victims compared with control cases. In this regard, Hsp70 expression was particularly evident not only in blood vessels but also in seromucosal secretory cells, ciliated epithelial cells, smooth muscle cells, and alveolar cells, suggesting a (supra-) vital response to hot vapor inhalation (10).

Another study confirmed a general tendency for high Hsp27 and Hsp70 expression levels in lung tissue, particularly in central airways, renal vasculature, and renal tubule cells, in fire fatalities. However, no differences in Hsp expression were observed depending on the burn degree. Therefore, Hsp27 and Hsp70 expression, particularly in lung and kidney tissues, may be used to determine vitality in fire or heat death. In particular, absent or low-level expression may have indicated the deceased was likely subjected to heat after death (11).

Furthermore, different Hsps may be used to estimate survival time since Hsp27 is expressed within seconds or minutes of a stressful exposure and in large amounts to protect cells, whereas Hsp70 takes up to 1 h to reach optimal expression levels (15).

### Hypothermia

In a study by Preuss et al. in 2008, approximately one fifth of fatal cases of hypothermia showed varying levels of Hsp70 expression in tubular epithelial cells and glomeruli in renal tissue, whereas the majority of control cases showed no or low Hsp70 expression. However, Hsp70 expression did not show a strong correlation with Wischnewski's spots (16).

A subsequent 2013 study did not confirm the findings that expression of Hsp70 is absent in control groups. Moreover, Hsp70 in glomerular podocytes indicated that expression was predominantly in the nucleolus, which appeared to be characteristic of hypothermia deaths. Therefore, analysis of Hsp70 expression patterns in glomeruli is potentially useful in forensic diagnostics to determine whether the ambient temperature was antemortem-low. A combination of immunohistochemical and real-time polymerase chain reaction (RT-PCR) studies also showed that Hsp70 was rapidly translocated to podocyte nuclei after cold exposure without new protein biosynthesis (17).

Besides renal tissue, pituitary gland showed slightly increased Hsp27 expression levels in hypothermia death cases compared with control cases, whereas Hsp70 showed no expression in either group. However, to identify hypothermia deaths, it is more appropriate to assess fatty degeneration by Sudan staining, which is used to assess hypothermia in almost half of the cases (18).

### Trauma

During traumatic injury to the frontal cortex, HSPA12B, the gene encoding HSP family A (Hsp70, member 12B), appears to be downregulated. Moreover, the combination of HSPA12B and FOSB gene expression diagnostically distinguished traumatic brain injury from control cases (mainly fatal cardiac events) (19). Additionally, HSPA7 and A13 gene transcripts appeared to be much higher in cases of traumatic injury than cases of mechanical asphyxia and sudden cardiac death (20).

### SIDS/Peripartum Deaths

Based on the hypothesis that SIDS may be associated with a decreased ability to respond to external stressors, a PCR analysis of Achilles tendon samples from SIDS cases indicated that HSPA1B (Hsp70) and HSPD1 (Hsp60) expression was increased in response to thermal stress. Furthermore, in SIDS cases where the infant was found in a prone position, lower HSPA1B expression was detected compared with cases where the infant was found on the side or the back (21). In contrast, an immunohistochemical study investigating the role of hyperthermia as a pathogenic factor for SIDS and Hsp27 expression analysis in the kidney, heart, and lung tissue revealed no meaningful differences between SIDS and control cases. Hsp70 was consistently negative in both groups and examined tissues (22).

An immunohistochemical study of brain and brainstem sections from 47 peripartum deaths showed increased Hsp70 and Hsp90 responses in the cytoplasm of neurons in non-acute cases of hypoxic-ischemic insults, whereas only mild responses were observed in isolated fields in acute cases. These observations could indicate that Hsp70 and Hsp90 are strongly expressed as later reactions in neurons (23).

### Excited Delirium

ED is one of several terms describing a syndrome characterized by delirium, agitation, and combativeness. HSPA1B transcript (Hsp70) expression was increased 1.8- to 4-fold in post-mortem brain tissue samples of ED cases. The mean core body temperature of cases, when recorded, was 40.7°C. Elevated Hsp70 levels in autopsy brain specimens may be considered to confirm that hyperthermia was an associated symptom, and often a forerunner, of death in these cases. Thus, a two-protein biomarker signature (in this study, HSPA1B and dopamine transporter levels) may serve as reliable forensic tools to identify ED at autopsy (24).

A later study addressing Mash et al. (24), quantified HSPA1A and HSPA1B gene transcript (encoding Hsp70) abundance in midbrain samples from a series of cocaine-related deaths and corresponding drug-free controls. Hsp70 expression was significantly increased in the cocaine-dependent group compared with controls, whether or not an ED was present. Elevated Hsp70 expression levels were predictive of a documented survival period between cocaine-use and death that included medical and/or police intervention, regardless of the presence of the ED syndrome. This study suggested that elevated Hsp70 expression was more likely to be related to survival times after drug use and/or medical and police intervention than to the presence or absence of ED *per se* (25).

### Drowning

Hsp70 expression in the neurons of the brain stem hypoglossal nucleus appeared to be significantly higher in drowning cases than other causes of asphyxia (hanging, strangulation, suffocation, asphyxia, and respiratory failure), suggesting that drowning caused more severe damage to the neurons of the hypoglossal nucleus. Furthermore, no correlations were identified between the rate of immunoreactivity and the post-mortem interval or survival in these cases. Thus, immunohistochemical examination of the hypoglossal nucleus could provide useful information to determine the cause of asphyxia (26).

In the lung tissue of freshwater and saltwater drowning and control cases, no statistically significant differences in Hsp70 expression levels were detected between respective groups. Only one case out of 10 cases each for freshwater and saltwater drowning displayed strong Hsp70 expression (27).

### Other Research Areas

Immunohistochemical examination of kidneys from forensic autopsy cases where methamphetamine was detected showed positive staining for Hsp70 in approximately one fifth of the cases, with antemortal hyperthermia confirmed in two cases. This suggested that heat stress may have led to Hsp70 expression. Interestingly, in Hsp-negative cases, methamphetamine levels in the blood were on average twice as high as Hsp-positive cases and almost 13 times as high in urine (28).

Individuals who died from mechanical asphyxia had higher mRNA levels of HSPA2, encoding Hsp70 (member 2), in the occipital region of the cerebral cortex compared with individuals who died from a traumatic injury. Hence, Hsp mRNA levels, as potential forensic biological markers in the occipital lobe, may provide clues to the cause and progression of death (20).

In cases of acute lung injury, extracellular Hsp72, encoded by the HSPA1A gene, was present in plasma and pulmonary edema fluid. Further, extracellular Hsp72 levels were highest in pulmonary edema fluid of patients with acute lung injury and preserved alveolar fluid clearance (29).

The findings of Preusse-Prange et al. (30) may provide some perspective to the aforementioned studies. These authors investigated the effects of formalin treatment using various protein detection methods on some members of the Hsp70 superfamily (HSPA1A and HSPA8). Their western blot analyses of formalin-fixed tissues failed to reliably detect proteins in cerebral and cerebellar tissue samples. In contrast, reproducible detection by immunohistochemistry was possible even after 1 month of incubation. However, protein detectability decreased proportionally to the fixation time. Therefore, only samples with a known fixation time should be used, and a fixation time longer than 1 month may lead to false-negative results.

Sample preparation and fixation times may explain why some studies conclude contrary results, and why in some studies, no Hsp expression is detected. Especially with regard to study comparability, a uniform approach may be useful. While reviewing the studies, we observed that methodologies, if specified, differed considerably. In gene analysis studies, the methodology primarily stated that samples were frozen at −70°C (20, 29) or −80°C (13, 14, 19, 21) immediately after collection. In two studies, the storage temperature was not specified (24, 25), and in one study, samples were submerged in RNA stabilization solution (17).

In reviewing Preusse-Prange et al., differences were apparent in immunohistochemical studies, which may have significantly limited comparability in some cases. With regard to fixation times, they were only stated in three studies. Fixation times were 24 h (27), 48 h (23), and between a few days and several years (16). The remaining studies did not report a fixation duration for their samples.

We also observed differences with respect to formalin concentrations. Results from Preusse-Prange et al. were based on a 10% fixation concentration. This was used in only two studies (17, 23). One study used 4% formalin (27) and four studies used 8–10% formalin (11, 15, 16, 18). Four studies did not report the formalin concentration (10, 22, 26, 28). Even though formalin fixation times appeared to have a significant influence on Hsp stainability, especially in the case of a prolonged fixation, it remains unclear whether these statements can also be applied to other, especially lower, formalin concentrations. This would require further investigation. However, this factor should be accounted for and considered as a possible source of negative results.

## Conclusions

In this 21-year range of selected studies, few provided absolute numbers; however, they dealt with different topics exploring the forensic significance of Hsp. There was no single focus; approximately equal attention was paid to fire deaths, hypothermia, SIDS/peripartum deaths, drowning, ED, and cardiac deaths. The ratio of immunohistochemical and gene analysis studies was approximately equal. Hsp70 was by far the most studied protein, followed by Hsp27.

These studies yielded several interesting findings with potentially relevant implications for forensic science. In fire deaths, consistent results indicated that Hsp27 and Hsp70 were useful markers for detecting (supra-) vitality; moreover, these markers could be used to estimate survival time. Hsp70 determination in blood may be a suitable marker for acute myocardial infarction and may be an exciting forensic approach for unclear deaths with suspected myocardial infarction or coronary insufficiency. For hypothermic deaths, Hsp70 displayed characteristic expression patterns in the kidney, which may be used diagnostically. In addition, the combined assessment of HSPA12B and FOSB gene expression may distinguish traumatic brain injury from control cases. HSPA1B and HSPD1 showed increased expression in response to thermal stress in SIDS cases. In ED, increased Hsp70 expression was demonstrated in the brain although the underlying pathophysiology remained unclear. Yet, in methamphetamine-related deaths, antemortal heat stress may have led to increased Hsp70 expression in the kidneys. Furthermore, drowning cases showed significantly higher Hsp70 expression levels in the neurons of the nucleus hypoglossus of the brainstem compared with other fatal asphyxia events. In addition, increased HSPA2 mRNA levels were observed in the cerebral cortex during mechanical asphyxia compared with traumatic injury.

However, it was striking that there were few, if any, follow-up studies on the respective research areas. Furthermore, a uniform approach to future studies should be considered. This factor could relate in particular to formalin concentrations and fixation times. This approach would not only avoid potentially false-negative results but also significantly increase comparability between respective studies.

This mini-review was limited by the fact that only studies on human material were included. Emphasis was placed on studies where immediate practical application is possible and where the results may, at best, add value to criminal investigations or court proceedings. Therefore, animal or experimental studies were deliberately excluded.

Nevertheless, our mini review highlighted the significant potential of Hsps and the established Hsp investigations in the forensic field. Our review indicated that despite the few studies available, those that were selected were very interesting in their own right, and the identified research approaches/areas require further study. Furthermore, it should be noted that, according to the defined criteria, no studies could be found that dealt with Hsp expression in the skin from a forensic point of view. Thus, it represents a vastly unexplored and potentially promising area of research.

## Author Contributions

JP: conception and study design and drafting the manuscript. JP and AM: acquisition of data. JP, BM, AM, and ED: analysis and/or interpretation of data, revising the manuscript critically for important intellectual content, and approval of the version of the manuscript to be published. All authors contributed to the article and approved the submitted version.

## Conflict of Interest

The authors declare that the research was conducted in the absence of any commercial or financial relationships that could be construed as a potential conflict of interest.

## Publisher's Note

All claims expressed in this article are solely those of the authors and do not necessarily represent those of their affiliated organizations, or those of the publisher, the editors and the reviewers. Any product that may be evaluated in this article, or claim that may be made by its manufacturer, is not guaranteed or endorsed by the publisher.
